# Identification of the circRNA–miRNA–mRNA Regulatory Network in Pterygium-Associated Conjunctival Epithelium

**DOI:** 10.1155/2022/2673890

**Published:** 2022-11-08

**Authors:** Jianfeng Yu, Jiawei Luo, Pengfei Li, Xiaojuan Chen, Guowei Zhang, Huaijin Guan

**Affiliations:** Eye Institute, Affiliated Hospital of Nantong University, Medical School of Nantong University, Nantong 226001, China

## Abstract

To investigate the regulatory mechanism of pterygium formation, we detected differentially expressed messenger RNAs (DE-mRNAs) and differentially expressed circular RNAs (DE-circRNAs) in pterygium-associated conjunctival epithelium (PCE) and normal conjunctival epithelium (NCE). Genome-wide mRNA and circRNA expression profiles of PCE and NCE were determined using high-throughput sequencing. Bioinformatics analyses, including Gene Ontology (GO) analysis, Kyoto Encyclopedia of Genes and Genomes (KEGG) pathway analysis, gene set enrichment analysis (GSEA), and protein–protein interaction (PPI) analysis, were conducted. The microRNAs (miRNAs) interacting with the hub DE-mRNAs and DE-circRNAs were predicted and verified using real-time quantitative PCR (RT-qPCR). The data showed that there were 536 DE-mRNAs (280 upregulated and 256 downregulated mRNAs) and 78 DE-circRNAs (20 upregulated and 58 downregulated circRNAs) in PCE. KEGG enrichment analysis indicated that the DE-mRNAs were mainly involved in the following biological processes: IL-17 signalling pathway, viral protein interaction with cytokine and cytokine receptor, cytokine–cytokine receptor interaction, ECM-receptor interaction, and focal adhesion. The GSEA results revealed that the epithelial mesenchymal transition (EMT) process was significantly enriched in upregulated mRNAs. The pterygium-associated circRNA–miRNA–mRNA network was established based on the top 10 DE-circRNAs, 4 validated miRNAs (upregulated miR-376a-5p and miR-208a-5p,downregulated miR-203a-3p and miR-200b-3p), and 31 DE-mRNAs. We found that miR-200b-3p, as a regulator of *FN1*, *SDC2*, and *MEX3D*, could be regulated by 5 upregulated circRNAs. In addition, we screened out EMT-related DE-mRNAs, including 6 upregulated DE-mRNAs and 6 downregulated DE-mRNAs. The EMT-related circRNA–miRNA–mRNA network was established with the top 10 circRNAs, 8 validated miRNAs (upregulated miR-17-5p, miR-181a-5p, and miR-106a-5p, downregulated miR-124-3p, miR-9-5p, miR-130b-5p, miR-1-3p, and miR-26b-5P), and 12 EMT-related DE-mRNAs. We found that hsa_circ_0002406 might upregulate *FN1* and *ADAM12* by sponging miR-26b-5p and miR-1-3p, respectively, thus promoting EMT in pterygium. Briefly, the study provides a novel viewpoint on the molecular pathological mechanisms in pterygium formation. CircRNA–miRNA–mRNA regulatory networks participate in the pathogenesis of pterygium and might become promising targets for pterygium prevention and treatment.

## 1. Introduction

Pterygium is characterized by fibrovascular tissue hyperplasia from the bulbar conjunctiva towards the cornea, accompanied by foreign body sensation, dry eye, astigmatism, and visual impairment [1, 2]. Once pterygium covers the pupillary area, it will cause a significant sight-threatening complication. The prevalence rates of pterygium were 8.8% in South Korea, 9.84% in China, and 38.7% in Northwest Ethiopia [3]. Currently, surgical excision is the main therapeutic method for pterygium. Nevertheless, recurrence is common. Excision combined with conjunctival autografts or amniotic membrane grafts is used to decrease postoperative recurrence [4]. However, the pterygium recurrence rate is still 3.3%-16.7% for conjunctival autografts and 6.4%-42.3% for amniotic membrane grafts [4]. Therefore, elucidating the pathogenesis and molecular mechanisms of pterygium is of great significance for preventing pterygium growth and recurrence.

Previous studies have demonstrated that inflammation, epithelial mesenchymal transition (EMT), DNA repair, cell proliferation, cell migration, and angiogenesis contribute to the pathogenesis of pterygium [5, 6]. Recently, an increasing number of studies on noncoding RNAs have demonstrated that the mechanism of pterygium is complex [7, 8].

Noncoding RNAs (ncRNAs), such as circular RNAs (circRNAs) and microRNAs (miRNAs), are involved in the transcriptional regulation of gene expression. CircRNAs are covalently closed continuous loops. CircRNAs can inhibit miRNA functions by sponging the target miRNAs directly or indirectly. The miRNAs are single-stranded noncoding RNAs composed of 18-24 nucleotides. Previous studies have shown that miRNAs downregulate gene expression by suppressing target mRNA translation or promoting mRNA degradation [9]. As reported, miRNAs significantly contribute to pterygium development by interacting with pterygium-associated mRNAs [10–14]. Moreover, circRNAs acting as miRNA sponges are differentially expressed in pterygium in comparison to conjunctival tissues [15]. Therefore, we speculated that the endogenous RNA regulatory network may play a key role in pterygium formation and development. However, integrative analysis of the circRNA–miRNA–mRNA regulatory mechanism in pterygium remains lacking. Ascertaining the circRNA–miRNA–mRNA regulatory network in pterygium is essential for preventing pterygium formation, growth, and recurrence.

**Figure 1:**
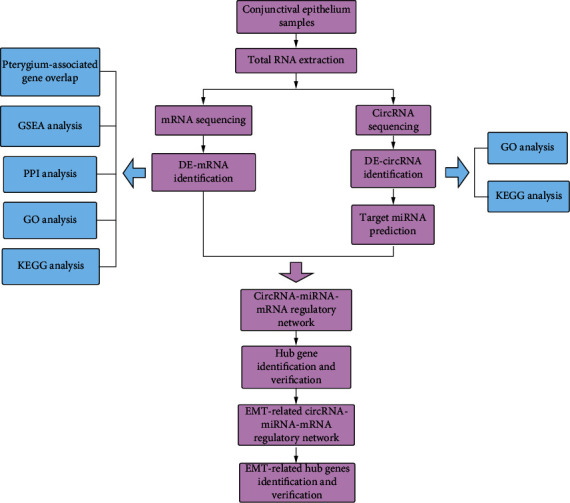
The workflow of study design.

In this study, as shown in Figure 1, we explored differentially expressed messenger RNAs (DE-mRNAs) and differentially expressed circRNAs (DE-circRNAs) in pterygium-associated conjunctival epithelium (PCE) and normal conjunctival epithelium (NCE) using high-throughput sequencing. Afterwards, the circRNA–miRNA–mRNA networks were established to help us understand the pathogenesis and underlying molecular mechanisms of pterygium.

## 2. Materials and Methods

### 2.1. Clinical Samples

The samples were obtained from the Department of Ophthalmology, the Affiliated Hospital of Nantong University. The inclusion criteria were primary nasal pterygium. Those included patients (four males and two females) aged from 49 to 79 years who underwent pterygium excision combined with conjunctival autografting. Three PCE samples were obtained from the nasal conjunctival epithelium in the pterygium. Three NCE samples were collected from the nasal conjunctival epithelium of donated eyes. The study was authorized by the Ethics Review Committee of the Affiliated Hospital of Nantong University and followed the Declaration of Helsinki. The participants signed the informed consent form. The samples were preserved at -80°C until further processing.

### 2.2. Preparation of Sequencing Libraries and Sequencing of mRNAs and circRNAs

Briefly, total RNA was purified from the samples with the RNeasy Kit (Qiagen, Duesseldorf, Germany). After qualification of RNAs using the NanoDrop One system (Waltham, MA, USA), the sequencing libraries were established by the Whole RNA-seq Lib Prep Kit (ABclonal, Shanghai, China). The sequencing libraries were prepared and quantified via a Qubit 3.0 fluorometer (Invitrogen, Carlsbad, USA). Finally, the library sequencing was performed by an Illumina NovaSeq sequencer (Illumina, CA, USA).

The Arraystar Super RNA Labeling Kit was used in circRNA amplification and fluorescent cRNA transcription. Next, the acquired cRNAs were transcribed into the Human circRNA Array (8 ×15K, Arraystar). Then, the circRNA expression level was detected with “mapped back-splicing junction reads per million mapped reads” after circRNA verification.

### 2.3. DE-mRNAs and DE-circRNAs Identification

The low-quality reads for base quality lower than 20 were eliminated by Cutadapt software (v1.9.1) [16]. Gene annotation and reference genome files were obtained from online databases (UCSC and NCBI). Next, HISAT2 software (v2.0.1) was used to align the clean data to the reference genome [17]. The transcripts were annotated and indexed for expression analysis. Then, we used an annotated file as a reference gene set and estimated the related gene expression using RSEM software (v1.2.15) [18]. The differential expression was analysed using the Bioconductor package DESeq2 [19]. The mRNAs or circRNAs with *P* < 0.05 and |log_2_(foldchange)| ≥ 1 were identified as DE-mRNAs or DE-circRNAs.

### 2.4. Functional Analysis of DE-mRNAs and DE-circRNAs

Gene Ontology (GO) analysis, including categories of biological process (BP), cellular component (CC), and molecular function (MF), and enrichment analysis of Kyoto Encyclopedia of Genes and Genomes (KEGG) pathway were performed to reveal the functions of DE-mRNAs and DE-circRNAs. Enrichment analysis of GO categories was performed by R “cluster Profiler” package, and enrichment analysis of KEGG pathways was tested upon hypergeometric distribution by R “phyper” function [20]. Those GO categories with adjusted *P* < 0.05 and KEGG pathways with *P* < 0.05 were considered as significantly enriched. Meanwhile, GO categories or KEGG pathways with less than 3 DEGs were discarded.

### 2.5. Protein–Protein Interaction (PPI) Analysis

The pathway database (http://www.pathwaycommons.org/) was used to analyse the PPI network in DE-mRNAs [21]. We selected direct interactions among DE-mRNAs. The R igraph package was applied to identify whether the genes were significant according to three attributes (event, betweenness, and degree) in the PPI network [22]. Genes involved in identical biological processes were grouped. The ggplot2 package was used to plot the distribution of gene attributes [23].

### 2.6. Gene Set Enrichment Analysis (GSEA)

To avoid omission of key pathways and hub mRNAs in DE-mRNAs, GSEA was performed. The analysis involved 1,000 permutations of the gene set. The following criteria were applied to verify the crucial pathways: |NES| ≥ 1 and nominal *P* < 0.05. GSEA was performed by R “fgsea” package [24].

### 2.7. Construction of circRNA–miRNA–mRNA Regulatory Networks

The miRNA categorical data were downloaded from the online database (http://www.mirbase.org). The circRNA–miRNA–mRNA networks were established with TargetScan (http://www.targetscan.org/vert_72/) and miRanda [25].

### 2.8. Verification of Hub miRNAs and Hub circRNAs

The expression of hub miRNAs that could interact with hub mRNAs and circRNAs was detected in the samples by real-time quantitative PCR (RT-qPCR). Total RNA was purified from those samples. In the case of miRNA PCR, the miRNA RT-qPCR Starter Kit (RiboBio, Guangzhou, China) was used to reverse-transcribe miRNAs following the manufacturer's instructions. Total miRNAs were reverse-transcribed to cDNAs. Then, PCR was performed using SYBR Green Master Mix (Vazyme, Nanjing, China) with miDETECT A Track miRNA primers (RiboBio, Guangzhou, China) in QuantStudio 5 Real-Time PCR Systems (Applied Biosystems, Foster City, CA, USA). Relative miRNA expression was normalized to *U6*. In the case of circRNA PCR, the reaction was carried out using the RiboBio circRNA qRT-PCR Starter Kit (RiboBio, Guangzhou, China) and SYBR Green Master Mix (Vazyme, Nanjing, China). Relative circRNA expression was normalized to *GAPDH* expression. Significant miRNAs and circRNAs were identified depending on previous reports and predicted results.

### 2.9. Statistical Analysis

The limma package of R 3.6.3 was applied for data standardization [26]. The DE genes were screened following a standardized expression matrix. Moreover, when |log_2_(foldchange)| ≥ 1 and *P* < 0.05, the expression levels of mRNAs and circRNAs were deemed to be significantly different. The pheatmap package and ggplot2 package in R 3.6.3 were used in volcano plots and heatmap construction [27]. *P* < 0.05 was considered to indicate statistical significance. Further analysis was performed using GraphPad Prism software 8.0.

## 3. Results

### 3.1. DE-mRNA Screening

**Figure 2:**
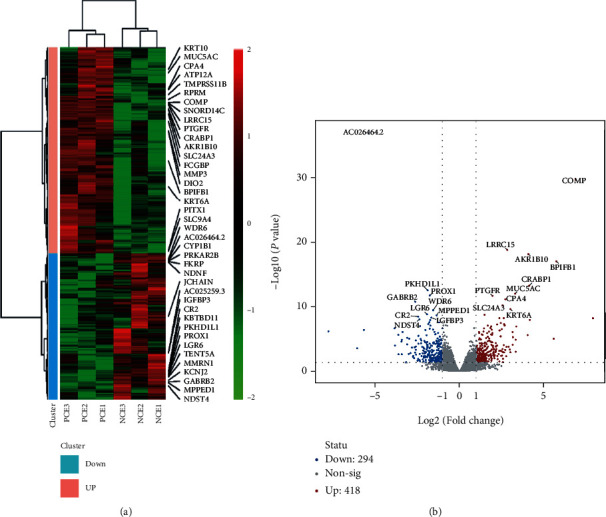
DE-mRNAs in PCE compared with NCE. (a) Heatmap of DE-mRNAs. The DE-mRNAs were clustered into two groups: upregulated mRNAs and downregulated mRNAs (PCE vs. NCE). (b) Volcano plot of all DE-mRNAs. The top 10 upregulated and downregulated DE-mRNAs are shown. Blue dots indicate the downregulated mRNAs, and red dots indicate the upregulated mRNAs.

The results showed that 19467 mRNAs were identified by high-throughput sequencing in PCE and NCE. A total of 536 mRNAs (|log_2_foldchange| ≥ 1, *P* < 0.05) were deemed to be differentially expressed. The mRNA expression patterns were shown using hierarchical clustering analysis (Figure 2(a)). Differences in mRNA expression between PCE and NCE were evaluated by volcano plot analysis (Figure 2(b)). Among those DE-mRNAs, 280 mRNA were upregulated, and 256 mRNAs were downregulated in PCE compared with NCE (Figure 2(b)).

### 3.2. Functional Analysis of DE-mRNAs

GO and KEGG analyses were used to reveal the underlying roles of DE-mRNAs.

**Figure 3:**
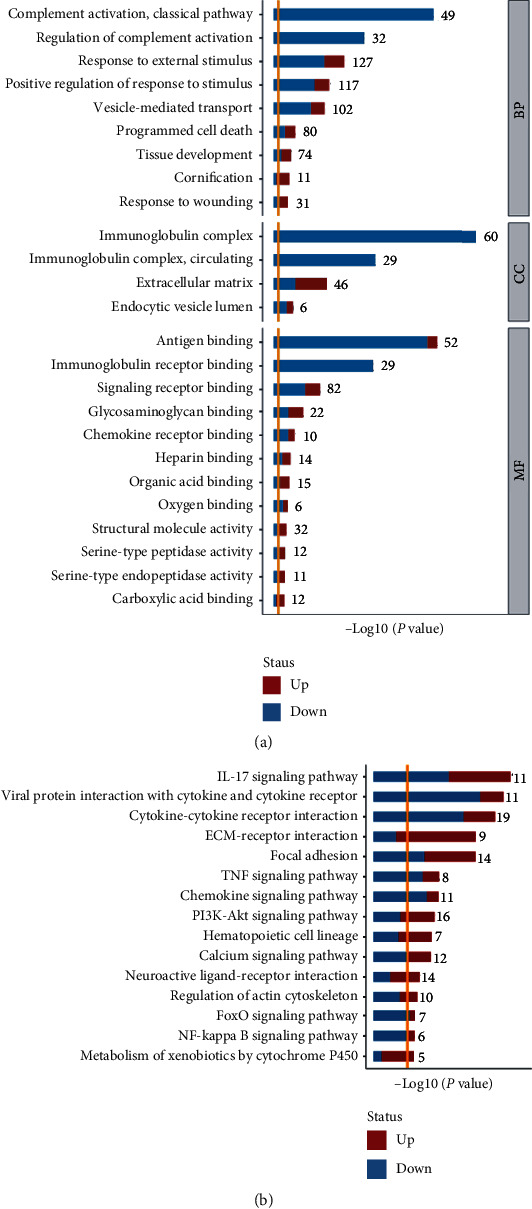
Gene Ontology enrichment and KEGG pathway analyses of DE-mRNAs. (a) Mainly enriched GO terms of DE-mRNAs. The GO terms were classified into three categories, including biological process (BP), cellular components (CC), and molecular function (MF). The lengths of the columns indicate the -log_10_(*P*) of input DE-mRNAs in GO terms. The number following the columns indicates the quantities of input DE-mRNAs in each GO term. The ratios of blue/red columns indicate the relative quantities of decreased DE-mRNAs vs. increased DE-mRNAs in each GO term. (b) The top 15 significantly enriched KEGG pathways. The lengths of the columns indicate the -log_10_(*P*) of input DE-mRNAs in KEGG pathways. The number following the columns indicates the quantities of input DE-mRNAs in each KEGG pathway. The ratios of blue/red columns indicate the relative quantities of decreased DE-mRNAs vs. increased DE-mRNAs in each pathway.

Among all DE-mRNAs, the significantly enriched BPs included complement activation classical pathway, regulation of complement activation, response to external stimulus, positive regulation of response to stimulus, and vesicle-mediated transport (Figure 3(a)). The main enriched CCs included immunoglobulin complex, immunoglobulin complex circulating, extracellular matrix, and endocytic vesicle lumen (Figure 3(a)). The main enriched MFs included antigen binding, immunoglobulin receptor, signalling receptor binding, glycosaminoglycan binding, and chemokine receptor binding (Figure 3(a)). The main enriched KEGG pathways of all DE-mRNAs are shown in Figure 3(b). The DE-mRNAs were primarily involved in the following pathways: IL-17 signalling pathway, viral protein interaction with cytokine and cytokine receptor, cytokine–cytokine receptor interaction, ECM-receptor interaction, focal adhesion, tumour necrosis factor (TNF) signalling pathway, chemokine signalling pathway, and PI3K-Akt signalling pathway (Figure 3(b)).

**Figure 4:**
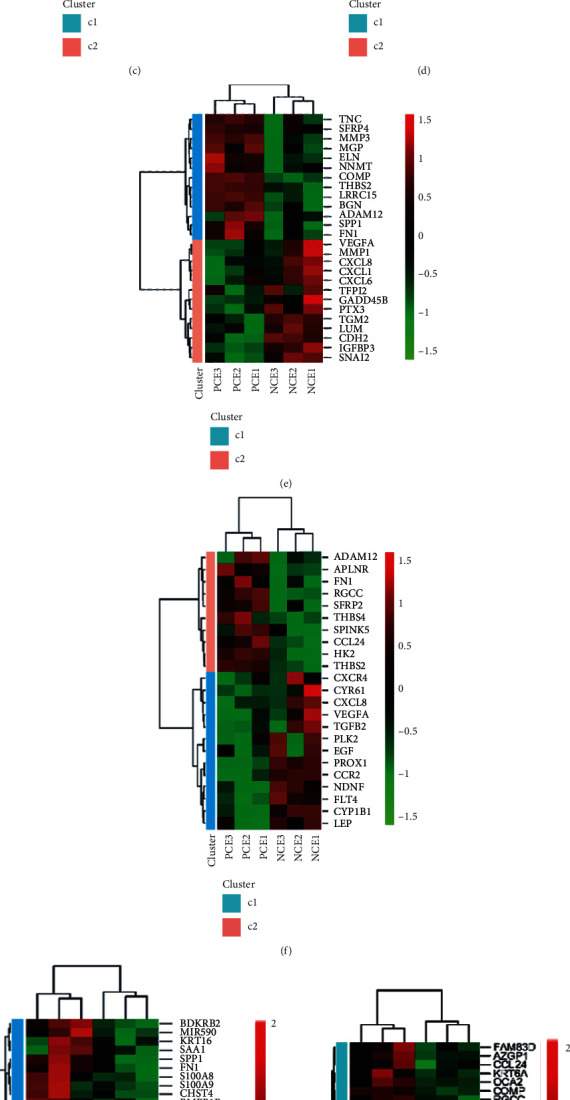
Identification of pterygium-associated DE-mRNAs. (a) Venn diagram was represented with 145 DE-mRNAs overlapping with pterygium-associated genes. (b) The overlapping DE-mRNAs were mainly involved in cell death, DNA repair, cell proliferation, EMT, inflammation, and angiogenesis. The columns indicate the input quantities of upregulated DE-mRNAs (red) and downregulated DE-mRNAs (blue). (c) Heatmap showing the cell death-related DE-mRNA expression. (d) Heatmap showing the DNA repair-related DE-mRNA expression. (e) Heatmap showing EMT-related DE-mRNA expression. (f) Heatmap showing inflammation-related DE-mRNA expression. (g) Heatmap showing angiogenesis-related DE-mRNA expression.

To further study the pathogenesis of pterygium, we overlapped the DE-mRNAs with pterygium-associated genes. We primarily screened for crucial genes involved in pterygium-associated biological processes (oxidative stress, DNA injury, DNA repair, inflammation, cell proliferation, autophagy, apoptosis, ferroptosis, angiogenesis, and so on). Briefly, 145 pterygium-associated DE-mRNAs were shown using a Venn diagram (Figure 4(a)). The two-dimensional histogram shows the numbers of DE-mRNAs in the processes of cell death, DNA repair, EMT, angiogenesis, inflammation, and cell proliferation (Figure 4(b)). Moreover, we showed pterygium-associated DE-mRNAs which were involved in the above pathophysiological processes (Figures 4(c)–4(h)).

### 3.3. GSEA of DE-mRNAs

**Figure 5:**
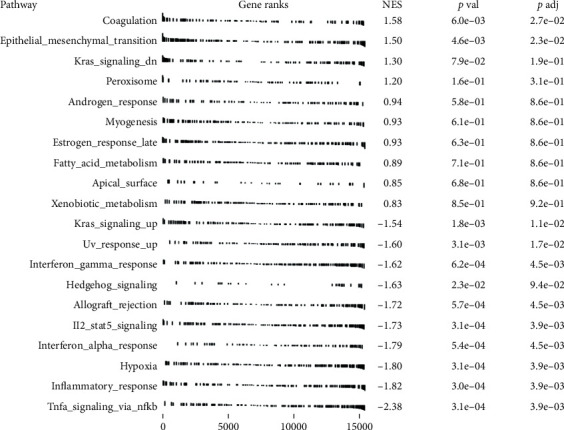
Gene set enrichment analysis of pterygium-associated DE-mRNAs. The functions of upregulated and downregulated DE-mRNAs were analysed by gene set enrichment analysis. The top 10 upregulated DE-mRNA enriched pathways (normalized enrichment score, NES ≥ 1, and *P* < 0.05) and top 10 downregulated DE-mRNA enriched pathways (NES ≤ −1 and *P* < 0.001) in PCE and NCE are shown. NES indicates the correlation and enrichment degree of DE-mRNA expression with pathways.

GSEA of upregulated and downregulated DE-mRNAs demonstrated that coagulation, EMT, kras-signalling-dn, and peroxisomes were significantly enriched in the upregulated DE-mRNAs (normalized enrichment score, NES ≥ 1, and *P* < 0.001) (Figure 5). The downregulated DE-mRNAs were involved in pathways including TNF-*α* signalling via NF-*κ*B, inflammatory reaction, hypoxia, interferon alpha (IFN-*α*) response, IL-2/STAT5 signalling, hedgehog signalling, and interferon gamma response (NES ≤ −1 and *P* < 0.05) (Figure 5).

### 3.4. PPI Network Analysis

**Figure 6:**
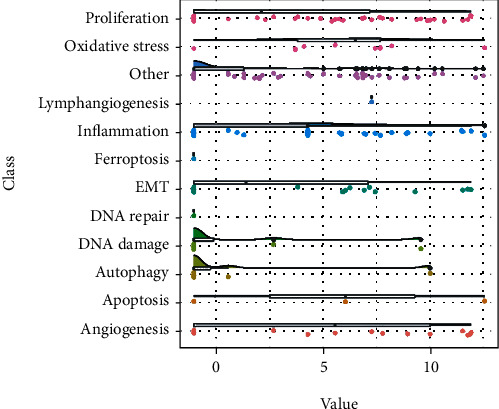
Raincloud plots of pterygium-associated DE-mRNAs. The *X* axis shows betweenness, and the *Y* axis shows pterygium-related biological processes. Dots indicate the position of gene betweenness, and curves indicate the density distribution of gene betweenness. This chart shows the distribution of the key pterygium-related DE-mRNAs in the PPI network.

The PPI network of DE-mRNAs was established (Figure 6). The PPI network revealed that the DE-mRNAs were involved in 12 key pterygium-associated pathways. The MCODE plugin was used to analyse the significant module (Figure 6).

### 3.5. Screening and Functional Annotation of circRNAs

**Figure 7:**
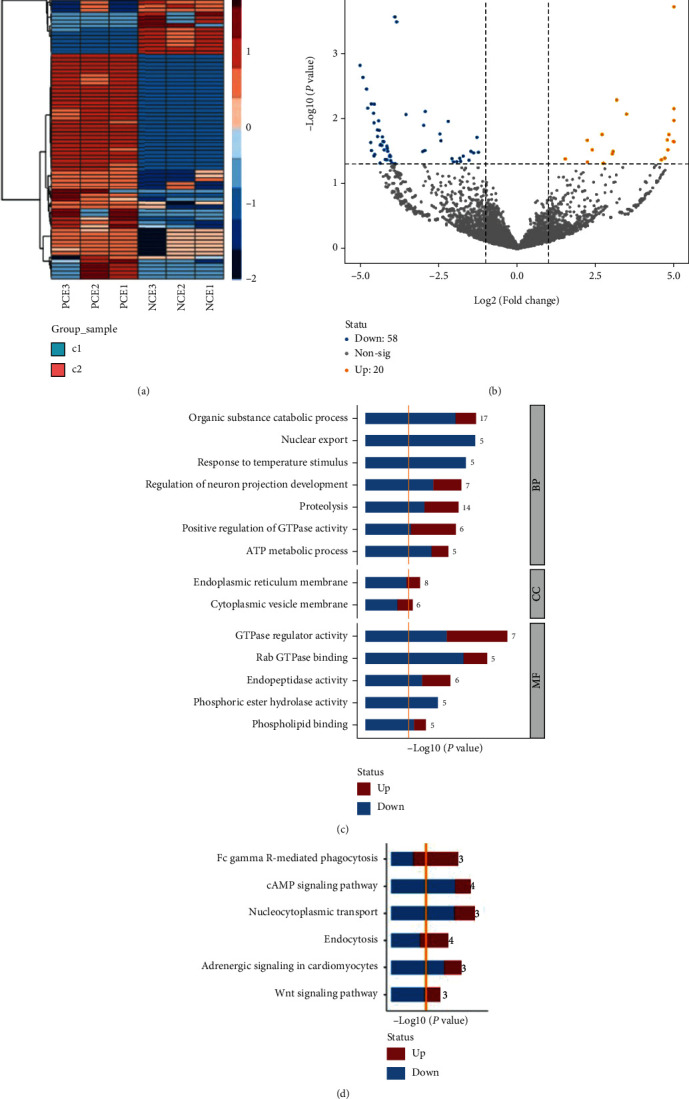
Identification and functional analysis of DE-circRNAs. (a) Heatmap of DE-circRNA expression in PCE and NCE. (b) Volcano plots of DE-circRNA expression in PCE and NCE. Blue dots indicate downregulated DE-circRNAs, and yellow dots indicate upregulated DE-circRNAs. (c) Main enriched BP, CC, and MF GO terms of DE-circRNAs. The lengths of the columns indicate the -log_10_(*P*) of input DE-circRNAs in GO terms. The number following the columns indicates the quantities of input DE-circRNAs in each GO term. The ratios of blue/red columns indicate the relative quantities of decreased DE-circRNAs vs. increased DE-circRNAs in each GO term. (d) The top 6 significantly enriched KEGG pathways of upregulated and downregulated DE-circRNAs. The lengths of the columns indicate the -log_10_(*P*) of input DE-circRNAs in KEGG pathways. The number following the columns indicates the quantities of input DE-circRNAs in each KEGG pathway. The ratios of blue/red columns indicate the relative quantities of decreased DE-circRNAs vs. increased DE-circRNAs input in each pathway.

CircRNAs can act as miRNA sponges to modulate gene expression [28]. To explore the potential pterygium-related circRNAs, we detected the circRNA expression profiles of PCE and NCE using high-throughput sequencing. The DE-circRNAs were clustered into two groups (Figure 7(a)). A volcano plot was used to filter DE-circRNAs (|log_2_(fold change)| ≥ 1, *P* < 0.05), revealing 20 upregulated and 58 downregulated DE-circRNAs in PCE compared with NCE (Figure 7(b)). GO analysis of DE-circRNA host genes was performed in three dimensions, including BP, CC, and MF. In the BP analysis, the main enriched categories included organic substance catabolic process, nuclear export, response to temperature stimulus, regulation of neuron projection development, and proteolysis (Figure 7(c)). In the CC analysis, the main enriched categories included the endoplasmic reticulum membrane and cytoplasmic vesicle membrane (Figure 7(c)). In the MF analysis, the main enriched categories included GTPase regulator activity, Rab GTPase binding, endopeptidase activity, phosphoric ester hydrolase activity, and phospholipid binding (Figure 7(c)).

### 3.6. Prediction and Validation of the circRNA–miRNA–mRNA Regulatory Network

**Figure 8:**
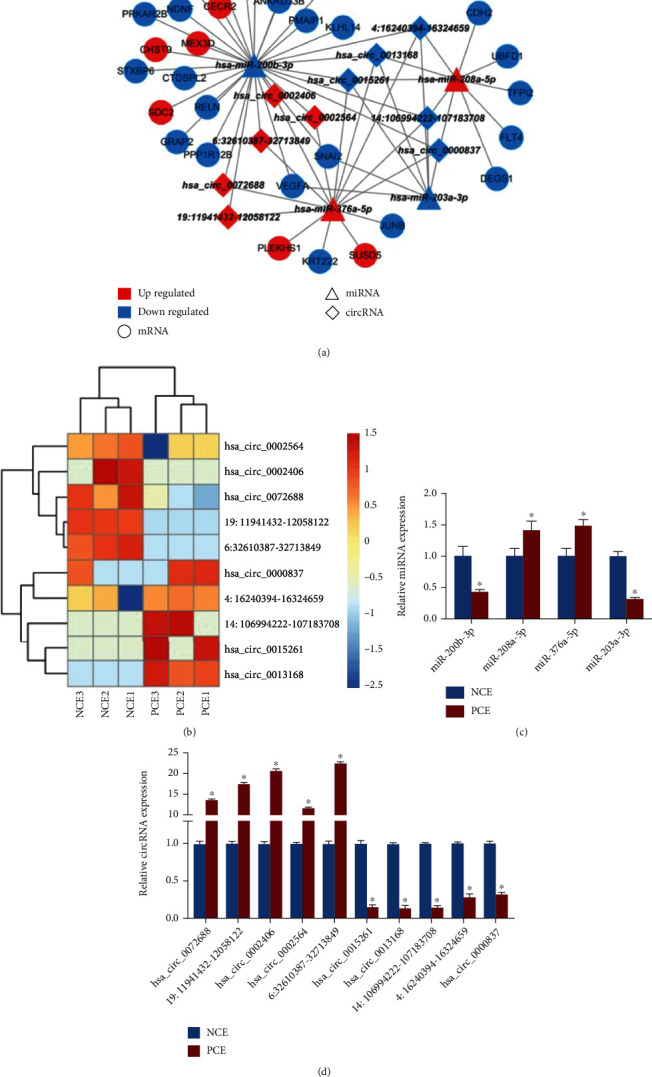
circRNA–miRNA–mRNA regulatory network. (a) The circRNA–miRNA–mRNA regulatory network was established. The top 5 upregulated/downregulated DE-circRNAs and top 10 DE-mRNAs were included in the network, and miRNAs interacting with circRNAs were predicted by miRBase. Blue dots indicate downregulated RNAs, and red dots indicate upregulated RNAs. The connecting lines indicate that interactions exist between each pair of RNAs. (b) Heatmap of hub DE-circRNAs in the network. (c) The expression of hub DE-miRNAs in PCE and NCE was verified by RT-qPCR. ^∗^*P* < 0.05. (d) The expression of hub DE-circRNAs in PCE and NCE was verified by RT-qPCR. ^∗^*P* < 0.05.

The circRNA–miRNA–mRNA regulatory network was analysed by miRanda software. The top 5 upregulated circRNAs and top 5 downregulated circRNAs were included in the network, and each circRNA bound to at least four miRNAs (Figure 8(a)). The top 5 upregulated/downregulated circRNAs were shown in the heatmap (Figure 8(b)). Next, we validated the expression of the top 5 upregulated/downregulated circRNAs and 4 predicted miRNAs in the regulatory network using RT-qPCR. It was demonstrated that miR-376a-5p and miR-208a-5p were significantly upregulated, whereas miR-203a-3p and miR-200b-3p were significantly downregulated in PCE (Figure 8(c)). The PCR results also showed that hsa_circ_0072688, 19:11941432-12058122, hsa_circ_0002406, hsa_circ_0002564, and 6:32610387-32713849 were significantly upregulated, whereas hsa_circ_0015261, hsa_circ_0013168, 14:106994222-107183708, 4:16240394-16324659, and hsa_circ_0000837 were significantly downregulated in PCE (Figure 8(d)).

### 3.7. Prediction and Validation of the EMT-Related circRNA–miRNA–mRNA Regulatory Network

**Figure 9:**
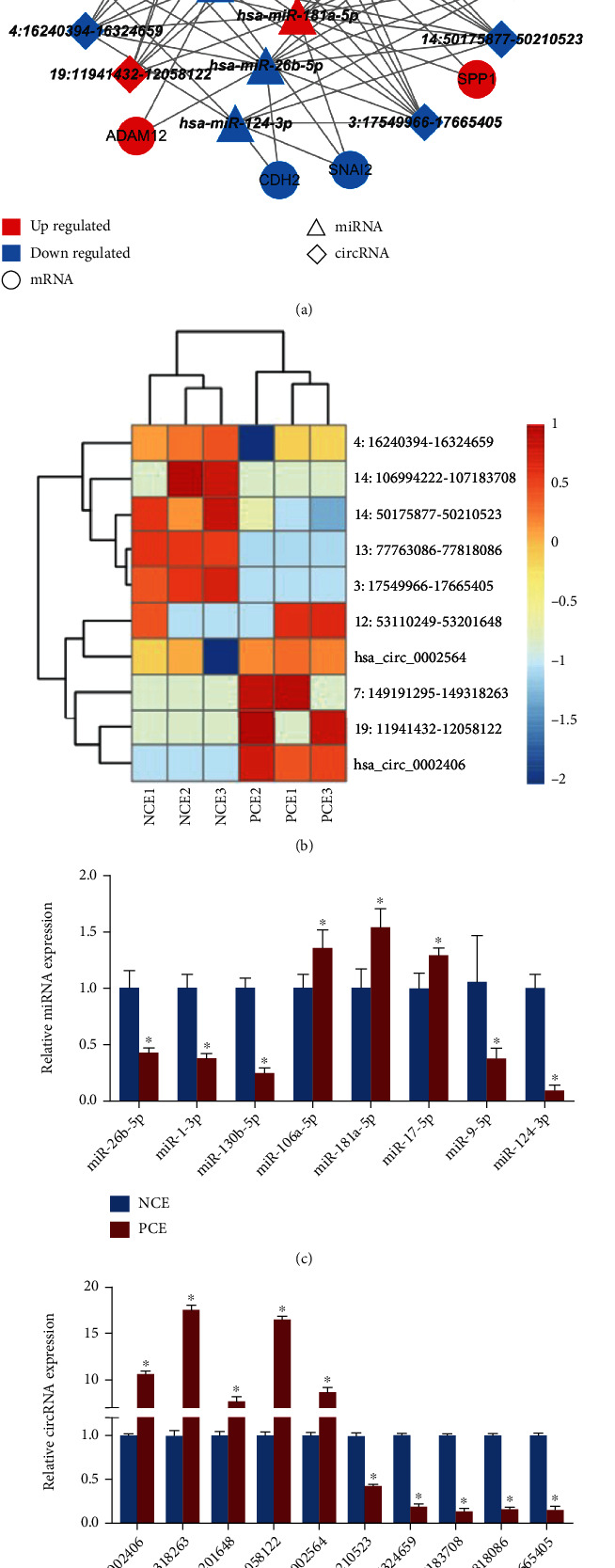
The EMT-related circRNA–miRNA–mRNA regulatory network. (a) The EMT-related circRNA–miRNA–mRNA network. This interaction network contains the top 10 DE-circRNAs, 8 predicted miRNAs, and 12 DE-mRNAs involved in the EMT process. Blue dots indicate downregulated RNAs, and red dots indicate upregulated RNAs. The connecting lines indicate that interactions exist between each pair of RNAs. (b) Heatmap of key EMT-related circRNAs in PCE and NCE. (c) The expression of key EMT-related miRNAs in PCE and NCE was verified by RT-qPCR. ^∗^*P* < 0.05. (d) The expression of key EMT-related circRNAs in PCE and NCE was verified by RT-qPCR.^∗^*P* < 0.05.

Recent studies have indicated that EMT in the conjunctival epithelium plays a crucial role in the pathogenesis of pterygium [13]. Therefore, we constructed an EMT-related circRNA–miRNA–mRNA regulatory network using miRBase (Figure 9). The EMT-related regulatory network contained 10 circRNAs (top 5 upregulated and top 5 downregulated circRNAs), 8 miRNAs, and 12 EMT-related mRNAs (Figure 9(a)). The heatmap of the top 5 upregulated/downregulated circRNAs in the EMT-related network were shown in Figure 9(b). The RT-qPCR results showed that the expression levels of miR-17-5p, miR-181a-5p, and miR-106a-5p were significantly upregulated, whereas the expression levels of miR-124-3p, miR-9-5p, miR-130b-5p, miR-1-3p, and miR-26b-5P were significantly decreased in PCE compared to NCE (Figure 9(c)). The PCR results also showed that hsa_circ_0002406, 7:149191295-149318263, 12:53110249-53201648, 19:11941432-12058122, and hsa_circ_0002564 were significantly upregulated, whereas 14:50175877-50210523, 4:16240394-16324659, 14:106994222-107183708, 13:77763086-77818086, and 3:17549966-17665405 were significantly downregulated in PCE (Figure 9(d)).

## 4. Discussion

Pterygium is a common ocular disease characterized by hyperplasia of conjunctival tissues on the cornea. The pterygium is mainly composed of vessels, fibroblasts, and epithelium. The pathogenesis and molecular mechanism of pterygium are still unclear. It was reported that EMT plays a key role in the pathogenesis of pterygium [29, 30].

Previous studies have focused on pterygium tissues (including pterygium epithelium and stroma); however, pterygium-associated EMT mainly occurs in pterygium epithelium. Therefore, we carefully dissected and sequenced the pterygium epithelium instead of the entire pterygium tissue. Moreover, previous studies have identified the roles of the lncRNA–miRNA network in pterygium, but very few studies have explored the circRNA–miRNA–mRNA regulatory network [7, 8]. In this study, we established the circRNA–miRNA–mRNA network in pterygium epithelium and provided deeper understanding of pterygium. The workflow of study design is shown in Figure 1.

The results showed that 280 DE-mRNAs were upregulated and 256 DE-mRNAs were downregulated in PCE compared with NCE. Among those DE-mRNAs, matrix metallopeptidase 3(*MMP-*3), fibronectin 1 (*FN1*), tenascin C (*TNC*), *LRRC15*, *KRT6A*, *COMP*, AKR1B10, and *MUC*5*AC* were significantly upregulated, which was consistent with previous studies on pterygium [8, 31–33]. The top 10 downregulated DE-mRNAs included *PKHD*1*L*1, *PROX*1, *GABRB*2, *WDR*6, *KCNJ*2, *LGR*6, *IGFBP*3, *MPPED*1, *CR*2, and *NDST*4. *MMP-*3 can promote ECM remodelling by activating other *MMPs*, including *MMP-*1, *MMP-*7, and *MMP-*9 [34]. Increasing evidence has demonstrated that *MMP-*3 upregulation in the conjunctival epithelium plays an important role in pterygium development [35]. FN1 is also an important molecule involved in the pathogenesis of pterygium that induces pterygium cell adhesion and migration [36].

Functional enrichment analysis demonstrated that DE-mRNAs were significantly enriched in the PI3K-Akt signalling pathway, ECM receptor interaction process, and haematopoietic cell lineage process, corresponding to previous studies [8, 37]. Bioinformatics analysis also indicated that EMT-related processes were significantly enhanced in PCE. It has been demonstrated that pterygium formation is closely connected with EMT of the conjunctiva [35]. Conjunctival epithelial cells can transform into pterygium stromal cells via the EMT process [38]. Moreover, EMT is also an important mechanism contributing to the migration and invasion of pterygium cells [39]. In this study, we found that EMT-related gene expression was significantly increased in the pterygium epithelium. To further investigate the pathway, we assessed the enrichment of DE-mRNAs in the EMT-related pathways. Among the EMT-related DE-mRNAs, *MMP-3*, *FN1*, *TNC*, and *NNMT* were significantly upregulated in PCE.

Studies have demonstrated that noncoding RNAs are involved in regulating pathogenic genes in pterygium [8, 40]. In the present research, we found that 20 DE-circRNAs were upregulated and 58 DE-circRNAs were downregulated in PCE compared with NCE. After predicting miRNAs interacting with circRNAs, we established the circRNA–miRNA–mRNA network, including the top 10 DE-circRNAs and top 10 DE-mRNAs. In addition, we verified 2 upregulated DE-miRNAs (miR-376a-5p and miR-208a-5p) and 2 downregulated DE-miRNAs (miR-203a-3p and miR-200b-3p) in the regulatory network using qRT-PCR. It has been previously discovered that miR-200b-3p was downregulated in pterygium as a regulator of *FN*1 [7, 8]. *MEX3D* and *SDC*2 were also important targets of miR-200b-3p. It was reported that *MEX3D* could promote proliferation of cervical carcinoma and transformation of prostatic epithelium in prostate cancer [41, 42]. *SDC*2 is involved in the EMT process of prostate cancer [43]. Moreover, we found that miR-200b-3p was negatively associated with five upregulated circRNAs, including hsa_circ_0002406, hsa_circ_0002564, hsa_circ_0072688, 6:32610387-32713849, and 19:11941432-12058122. We speculated that these highly expressed circRNAs may increase the expression of *FN1*, *SDC2*, and *MEX3D* by sponging miR-200b-3p and further promote pterygium formation and growth.

Since EMT is a key mechanism of pterygium, we also constructed an EMT-related circRNA–miRNA–mRNA network involving 10 DE-circRNAs (5 upregulated and 5 downregulated DE-circRNAs), 8 validated DE-miRNAs, and 12 DE-mRNAs. We found that the downregulation of miR-1-3p and miR-26b-5p could simultaneously induce the upregulation of *FN1* and *ADAM12*. Previous studies have demonstrated that *ADAM12* can promote tumour invasion and EMT [44, 45]. *FN1* has been reported in pterygium, as mentioned above. In addition, hsa_circ_0002406 was significantly upregulated and found to target seven miRNAs, including miR-1-3p and miR-26b-5p. Therefore, we speculated that hsa_circ_0002406 might promote the EMT process by sponging miR-1-3p/miR-26b-5p and then upregulating *FN1* in pterygium epithelium. The functions of hsa_circ_0002406 in pterygium have not been reported previously and need to be further studied.

Additionally, 5 upregulated circRNAs, including hsa_circ_0002406, 7:149191295-149318263, 12:53110249-53201648, 19:11941432-12058122, and hsa_circ_0002564, showed similar expression trends with *TNC* and inverse trends with miR-9-5p in the EMT-related circRNA–miRNA–mRNA network. TNC protein is an extracellular matrix glycoprotein that is critical for the process of EMT and cell proliferation [46]. It was reported that *TNC* mRNA is a target of miR-9-5p [47] and is involved in pterygium development [8]. These upregulated circRNAs may increase *TNC* expression by targeting miR-9-5p.

In conclusion, we identified DE-mRNAs and DE-circRNAs in PCE using high-throughput sequencing and then established circRNA–miRNA–mRNA regulatory networks. We found that circRNAs may regulate EMT-related genes by targeting miRNAs and play an important role in pterygium. The limitation of this study is that the content of circRNA databases is not sophisticated enough, and we could not fully elucidate the functions of all DE-circRNAs. In the future, we will further verify the regulatory functions of filtered key DE-circRNAs and elucidate the underlying molecular mechanisms in pterygium occurrence and growth.

## Data Availability

The data that support the findings of this study are available from the corresponding author upon reasonable request.
